# Multi-omics analysis reveals genes and metabolites involved in *Bifidobacterium pseudocatenulatum* biofilm formation

**DOI:** 10.3389/fmicb.2023.1287680

**Published:** 2023-11-09

**Authors:** Ting Zhang, Zongmin Liu, Hongchao Wang, Hao Zhang, Haitao Li, Wenwei Lu, Jinlin Zhu

**Affiliations:** ^1^State Key Laboratory of Food Science and Resources, Jiangnan University, Wuxi, China; ^2^School of Food Science and Technology, Jiangnan University, Wuxi, China; ^3^(Yangzhou) Institute of Food Biotechnology, Jiangnan University, Yangzhou, China; ^4^National Engineering Research Center for Functional Food, Jiangnan University, Wuxi, China

**Keywords:** *Bifidobacterium pseudocatenulatum*, biofilm, multi-omics, stress response, EPS

## Abstract

Bacterial biofilm is an emerging form of life that involves cell populations living embedded in a self-produced matrix of extracellular polymeric substances (EPS). Currently, little is known about the molecular mechanisms of *Bifidobacterium* biofilm formation. We used the *Bifidobacterium* biofilm fermentation system to preparation of biofilms on wheat fibers, and multi-omics analysis of both *B. pseudocatenulatum* biofilms and planktonic cells were performed to identify genes and metabolites involved in biofilm formation. The average diameter of wheat fibers was around 50 μm, while the diameter of particle in wheat fibers culture of *B. pseudocatenulatum* was over 260 μm at 22 h with 78.96% biofilm formation rate (BR), and the field emission scanning electron microscopy (FESEM) results showed that biofilm cells on the surface of wheat fibers secreted EPS. Transcriptomic analysis indicated that genes associated with stress response (*groS*, *mntH*, *nth*, *pdtaR*, *pstA*, *pstC*, *radA*, *rbpA*, *whiB*, *ybjG*), quorum sensing (*dppC*, *livM*, *luxS*, *sapF*), polysaccharide metabolic process (*rfbX*, *galE*, *zwf*, *opcA, glgC*, *glgP*, *gtfA*) may be involved in biofilm formation. In addition, 17 weighted gene co-expression network analysis (WGCNA) modules were identified and two of them positively correlated to BR. Metabolomic analysis indicated that amino acids and amides; organic acids, alcohols and esters; and sugar (trehalose-6-phosphate, uridine diphosphategalactose, uridine diphosphate-N-acetylglucosamine) were main metabolites during biofilm formation. These results indicate that stress response, quorum sensing (QS), and EPS production are essential during *B. pseudocatenulatum* biofilm formation.

## 1. Introduction

There are different forms of bacterial life such as sessile or surface-free forms of life. One of them is planktonic, and another is microbial aggregates, which is also called biofilm (Sauer et al., 2022). The formation stages of biofilms on biological or abiotic surfaces included five steps: reversible attachment, irreversible attachment, maturation I, maturation II, and dispersion (Sauer et al., 2022). The reversible attachment stage is started when bacteria reach surfaces successfully. Some bacteria with weak adhesion return to the liquid, while others can produce stronger adhesion and enter the irreversible attachment stage. In this stage, many genes related to adhesion are activated. For example, *algC*, genes involved in the biosynthesis of the Psl matrix polymer, and genes linked to antibiotic resistance are activated in the irreversible attachment stage of *Pseudomonas aeruginosa* biofilm (Gupta et al., 2013). In addition, EPS are produced in this stage. EPS includes water-soluble polysaccharides, proteins, eDNA, and water-soluble compounds (Flemming et al., 2023). EPS generated in the biofilm evolves at different stages, and this evolution is a response to available nutrients and the environmental conditions (Flemming et al., 2023). After the bacterial adhesion is stable, the formation of biofilm enters to mature stage. QS plays an important role in this process. QS is a cell-to-cell chemical communication process. When the cell density reaches a threshold level, QS might be activated, which promotes the switch to a biofilm lifestyle in a coordinated manner (Solano et al., 2014). QS signal molecules commonly have a low molecular weight, including acyl homoserine lactones (AHLs) (Fekete et al., 2007), *cis*-2-unsaturated fatty acids (Zhou et al., 2017), modified oligopeptides (also called autoinducing peptides, AIPs) (Verbeke et al., 2017), and furanosyl-borate diesters (autoinducer-2, AI-2) (Zhang et al., 2020). Dispersion is the last stage of biofilm. In this stage, bacteria leave the matrix and return to planktonic module, and dispersion is consistent with the reduction and degradation of matrix components (Rumbaugh and Sauer, 2020). Although the stages of biofilm formation have been clearly divided into five stages, there is still a lot of researches needed to investigate the genes, metabolites, and mechanisms involved in the biofilm formation for different bacteria.

Biofilm provides a protective ecological niche for cells in nature, helping them escape host defense. Therefore, biofilm is widely present in environments such as water (Pronk et al., 2019), soil (Vlamakis et al., 2013), and food (Lu et al., 2022). In addition, researchers have found that a large number of gut bacteria can form biofilms. The biofilm dominated by *Bacteroides fragilis* is believed to be related to the formation of inflammatory bowel disease (IBD), and the average density of mucosal biofilm in IBD patients is 100 times higher than that in patients with irritable bowel syndrome or healthy subjects (Swidsinski et al., 2005). *Clostridium difficile* is the most common pathogen of antibiotic associated diarrhea, and one of the reasons for its resistance is the formation of biofilms (Peng et al., 2017). In addition to pathogenic bacteria that can form biofilms, probiotics also exhibit biofilm forming ability. The biofilm formed by *Lactobacillus rhamnosus* and *Lactobacillus plantarum* showed resistance to antibiotics. Incorporating probiotic biofilm into yogurt can enhance the vitality of probiotic strains (Rezaei et al., 2021). As the first batch of gut colonizing bacteria, *Bifidobacterium* have also received attention for their biofilm forming ability. Six *Bifidobacterium* (*B. adolescents*, *B. bifidum*, *B. animalis*, *B. longum*, *B. breve*, and *B. pseudocatenulatum*) can form biofilms on the rough and porous surface of grape seeds flour, and the six bacteria exhibited different biofilm formation ability (Liu et al., 2021a). Due to the complex environment of the intestine, the study of gut microbiota membrane formation still needs to consider the impact of environmental stress (Sánchez et al., 2013). Under 3% oxygen treatment, *B. longum* can induce oxidative stress and facilitate the formation of biofilms. High concentration of bile may lyse *Bifidobacterium* cells and release QS signaling molecules to make *Bifidobacterium* adhere (Kelly et al., 2020). It can be seen that environmental conditions are also a necessary factor to consider when studying the biofilm formation of bacteria. In addition to environmental impacts, the formation of biofilms is also highly controlled by genetic level (Toyofuku et al., 2016). For instance, QS signaling molecules, cyclic adenosine monophosphate (cAMP), bis-(3′-5′)-cyclic diguanosine monophosphate (c-di-GMP), and two-component systems (TCS) (Liu et al., 2019, 2020; Liu X. et al., 2022). The *luxS* gene was discovered during the formation process of *B. longum* NCC2705 biofilm (Kaufmann et al., 2014). This gene enabled *B. longum* NCC2705 to produce AI-2 activity and exhibited regulatory functions in biofilm formation. The elevated expression levels of *cyaA* and Bbi37 | peg.1398 (cAMP receptor) have been observed during the biofilm formation process of *B. bifidum*. cAMP produced by adenylate cyclase *cyaA* can regulate the Tad IV pili, which is the core of the surface sensing mechanism in the early stage of biofilm formation (Liu et al., 2021b). Interestingly, through comparative genomics techniques, it has been found that *B. pseudocatenulatum* contain c-di-GMP pathway, which plays an important role in biofilm formation (Liu Z. et al., 2022). However, the formation process and regulatory mechanisms of *B. pseudocatenulatum* biofilm have not been thoroughly explored.

Omics technology has been widely applied to analyze the formation mechanism of biofilms. Compared to single-omics analysis, multi-omics analysis can provide more comprehensive information from multi-level results such as genes, mRNA levels, protein expression and regulation, and cell metabolism (Rizo et al., 2020). For example, when exploring the preventive effect of *Lactiplantibacillus plantarum* CCFM8724 on dental caries, a combination of metabolomics and transcriptomics analysis found that the supernatant of *L. plantarum* CCFM8724 had a positive response to glucose metabolism in the biofilm of a mixture of *Streptococcus mutans* and *Candida albicans*, and inhibiting the expression of virulence genes in *C. albicans* (Zhang et al., 2021). Through multi-omics analysis, it was found that thymoquinone disrupted the QS system of *Pseudomonas aeruginosa*. The dysfunctional QS and inhibited antioxidant enzymes lead to enhanced oxidative stress, which in turn disrupts the energy and protein metabolism of *P. aeruginosa* PAO1 (Chen et al., 2022).

This study utilized multi-omics techniques to analyze key genes and metabolites involved in the formation of *Bifidobacterium* biofilms. This article focused on *B. pseudocatenulatum* as the research object, with wheat fibers as the biofilm-forming substrate. A dynamic fermentation system was used to prepare biofilms, and transcriptome and metabolomic analyses were conducted on the biofilm and planktonic cells. This study aims to provide an analytical basis for studying the genes and metabolites that affect the key stages of biofilm formation in *Bifidobacterium*.

## 2. Materials and methods

### 2.1. Biofilm culture

Three *B. pseudocatenulatum* strains FJLHD4M2, FFJNDD6M2 and FHNBA14M1 used in this study were obtained from the Jiangnan University (Wuxi, China). These strains were grown anaerobically at 37°C in de Man, Rogosa and Sharpe (MRS) broth. Activated strains were inoculated into Erlenmeyer flask (4%, v/v) in a constant temperature incubator at 37°C and 120 rpm. Wheat fibers with an average diameter of 50 μm were added to culture media with 4% (w/v) for biofilm formation, and no wheat fibers added groups were used as the control groups (Iltis et al., 2011; Parrish and Fahrenfeld, 2019). In both wheat fibers groups (WF) and control groups (C), the bacteria were grown anaerobically in batch culture for a duration of 32 h without adding fresh growth medium.

The pH values and colony forming units (CFU) counting of wheat fibers culture groups and control groups were determined during fermentation (Ushakova et al., 2012). For obtaining the total cell number in wheat fibers cultures (WT), samples were dispersed by vortexing for 30 s, sonicating for 10 s, and vortexing again for 30 s (Grumezescu et al., 2014). For obtaining the planktonic cell number in wheat fibers cultures (WP), samples were centrifugated at 100 rpm for 2 min and took the supernatants. Using 0.9% NaCl solution to serial dilute WT and WP suspensions, then 0.1 mL diluted suspension was added to MRS solid plate and anaerobically incubated for 48 h. The BR = (CFU count in WT - CFU count in WP)/ CFU count in WT*100%. The size distribution of the three strains on wheat fibers at 22 h was determined by a laser particle size analyzer.

### 2.2. FESEM

To observe the structure of the biofilm formed on wheat fibers. The upper layer of fermentation liquid after dynamic fermentation were removed, and the precipitated particles were filtered by 40 μm cell sieves. Then, the intercepted particles were dehydrated with ethanol for 10 min. Before examine by FESEM, particles were coated with gold (Flemming et al., 2016).

### 2.3. RNA-Seq and reads mapping

Biofilms formed by all the three strains of *B. pseudocatenulatum* after 10 h, 22 h, and 32 h of growth (as described in 2.1) were considered for RNA isolation and sequencing. The HiPure HP Plant RNA Mini Kit was used for RNA isolation. RNA-sequencing was conducted using an Illumina HiSeq 4,000. Transcriptome data of *B. pseudocatenulatum* have been stored in the national center for biotechnology information (NCBI), and the BioProject code is PRJNA738670.^1^ fastp was used to preprocess paired-end reads (Vasquez et al., 2020). Reads were aligned to *B. pseudocatenulatum* FJLHD4M2 genomes using HISAT2 v2.20 (Kim et al., 2019). Raw read counts were created using featureCounts (Liao et al., 2014).

### 2.4. Untargeted metabolome

For metabolite extraction, the samples were centrifuged under 12,000 rpm at 4°C for 10 min, and the 100 μL supernatants were mixed with 800 μL of ice-cold mixture (methanol: acetonitrile: H_2_O = 2: 2: 1) for 15 min before lyophilization (Liu et al., 2021c). Finally, the dried samples were stored in −80° for liquid chromatograph-mass spectrometer (LC–MS) assay (Liu et al., 2021c).

The sample injection volume was 5 μL with 0.5 mL/min flow rate, and the column temperature was 40°C. The positive mode mobile phase was consisted by water (A) and acetonitrile (B) containing 0.1% (v/v) formic acid, while that of the negative mode was water (A) and acetonitrile (B) containing 1 mM ammonium fluoride and 0.1% formic acid. For both modes, the elution gradient (A: B, v/v) was as follows: 80:20 from 0 to 1 min, 0:100 at 7 min and kept 4 min, and then 80: 20 at 11.5 min and kept 2 min (Liu et al., 2021c). Raw data handling was done using compound discoverer software. MetaboAnalyst5.0^2^ was used for the metabolic pathway and function analysis (Favre et al., 2017; Wong et al., 2018).

### 2.5. Differential gene analysis

DESeq2 was used to analyze differential gene expressions at different biofilm formation stages (Love et al., 2014). Specified pairwise transcriptome comparisons (10 h WF vs. 10 h C, 22 h WF vs. 22 h C, 32 h WF vs. 32 h C) were performed to identify the main differentially expressed genes (DEGs) with an absolute value of log2 (fold change) > 1.0 (Kiu et al., 2020; Liu et al., 2021c). TBtools was used to draw UpSet graphs for displaying the comparison and overlap (Chen et al., 2020). Kyoto encyclopedia of genes and genomes (KEGG) pathway and gene ontology (GO) analyses were conducted using clusterprofiler (R package) (Yu et al., 2012).

### 2.6. PPI network analysis

The protein–protein interaction (PPI) network serves as a powerful tool to elucidate potential interactions between DEGs. In our study, these interactions were analyzed using the STRING database. To ensure specificity and relevance to our subject of interest, we restricted the species parameter to “*Bifidobacterium pseudocatenulatum*” and set an interaction score threshold of >0.4 to filter out low-confidence interactions. Visualization of the resultant PPI network was achieved using Cytoscape software version 3.8.0, providing a comprehensive graphical representation of how these DEGs potentially interact with one another in the context of biofilm formation (Karimizadeh et al., 2019).

### 2.7. Identification of WGCNA modules

WGCNA is a robust method that groups genes into modules based on their co-expression patterns, aiming to identify sets of genes that might be functionally related or co-regulated (Langfelder and Horvath, 2008). The construction of the WGCNA network and subsequent module detection were done using an unassigned type of topological overlap matrix. To optimize the network, a power β of 11 and a branch merge cut height of 0.25 were chosen based on recent studies (Han et al., 2020; Liu et al., 2021c). We then selected gene network files based on suitable WGCNA edge weight values to be input into Cytoscape, focusing specifically on the ‘Red’ and ‘Magenta’ modules with WGCNA edge weights exceeding 0.2. Within these modules, the MCODE tool in Cytoscape was employed to identify and highlight hub genes, which are central nodes in the network and play pivotal roles in biofilm formation.

### 2.8. Statistical analysis

The graphs of the data were drawn using the package ggplot2 (v3.3.2). The R package ComplexHeatmap (v2.5.1) was used to process the heatmap (Gu et al., 2016). The difference was calculated using one-way analysis of variance and considered statistically significant at *p* < 0.05 (Liu et al., 2021b).

## 3. Results

### 3.1. *Bifidobacterium pseudocatenulatum* formed biofilms on wheat fibers

**Figure 1:**
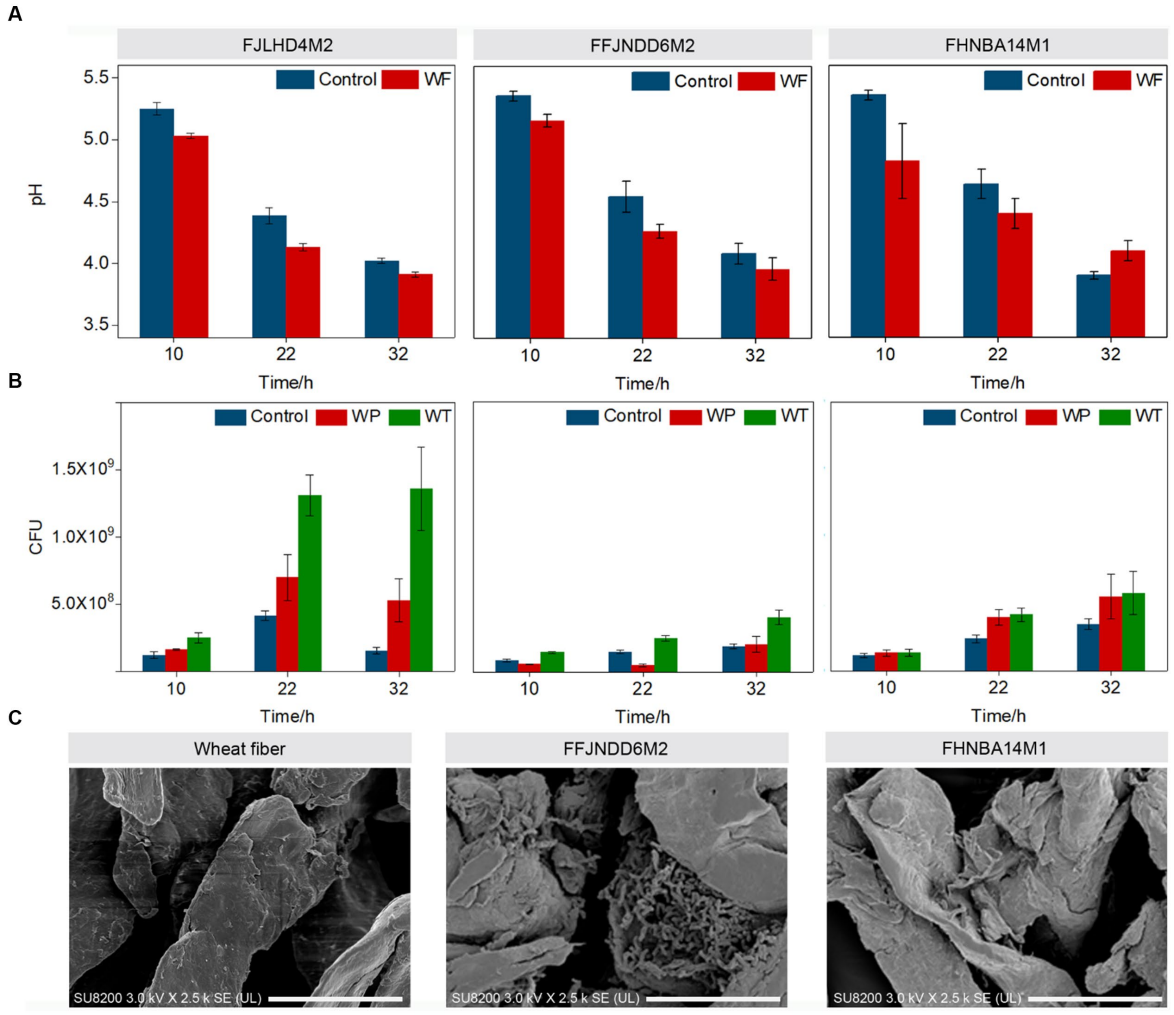
Biofilm formation of three *B. pseudocatenulatum* strains on wheat fibers. **(A)** The change of pH values at different biofilm formation stages in the wheat fibers (WF) and control cultures. **(B)** The CFU counts at different biofilm formation stages. WT: the total cell number in wheat fibers culture; WP: the planktonic cell number in wheat fibers culture. **(C)** The FESEM of wheat fibers, FFJNDD6M2 and FHNBA14M1 on wheat fibers at 22 h, 2,500×. Scan bar indicated 20 μm.

The pH values in the control and wheat fibers cultures of three *B. pseudocatenulatum* strains were decreased during 0–32 h (Figure 1A). The WT count recorded in the wheat fibers culture of FJLHD4M2, FFJNDD6M2, and FHNBA14M1 strains were 1.36 × 10^9^, 4.05 × 10^8^, and 5.80 × 10^8^ CFU/mL at 32 h, respectively (Figure 1B) in contrast to 1.56 × 10^8^, 1.90 × 10^8^, and 3.50 × 10^8^ CFU/mL at 32 h in the respective control cultures for each strain (Figure 1B). The BR is used to evaluate the level of biofilm formation, which can be calculated by CFU count in WT and WP. The higher the BR, the more bacteria adhere to the wheat fibers. The highest BR was recorded in FFJNDD6M2 strain at 22 h (Supplementary Table S1), and the corresponding CFU count in WT and control groups were 2.50 × 10^8^ and 1.48 × 10^8^, respectively. The BR of FHNBA14M1 was lower than 5% during the whole fermentation (Supplementary Table S1). Based on WT and WP, the biofilm formation process was divided to three stages. The early stage named S1 (10 h), at which bacteria were increasing on the wheat fibers. The maturation stage named S2 (22 h), at which the number of bacteria on the wheat fibers increases highest. The dispersion stage named S3 (32 h), where bacteria were leaving the wheat fibers. The FESEM results showed that *B. pseudocatenulatum* adhered to the surface of wheat fibers and secreted some extracellular substances (Figure 1C). The average diameter of wheat fibers was around 50 μm, but the diameter of 33.4% particles in wheat fibers culture of FFJNDD6M2 was over 260 μm at 22 h (Table 1). However, the average particle size in wheat fibers culture of FHNBA14M1 was around 50 μm (Table 1) with the BR lower than 5% (Supplementary Table S1) at 22 h, and a little cell attached on wheat fibers (Figure 1C).

**Table 1:** Particle size of wheat fibers (WF) and biofilms formed by three different *B. pseudocatenulatum* strains at 22 h.

Strains	Dia (μm)	Vol (%)	Width
WF	53.64	100.0	98.39
FJHD4M2	269.2	33.4	214.00
	51.18	66.6	78.90
FFJNDD6M2	152.8	58.0	207.80
	32.19	42.0	32.78
FHNBA14M1	54.21	100.0	99.61

### 3.2. Biofilm formation associated with stress response, quorum sensing, polysaccharide metabolic process

**Figure 2:**
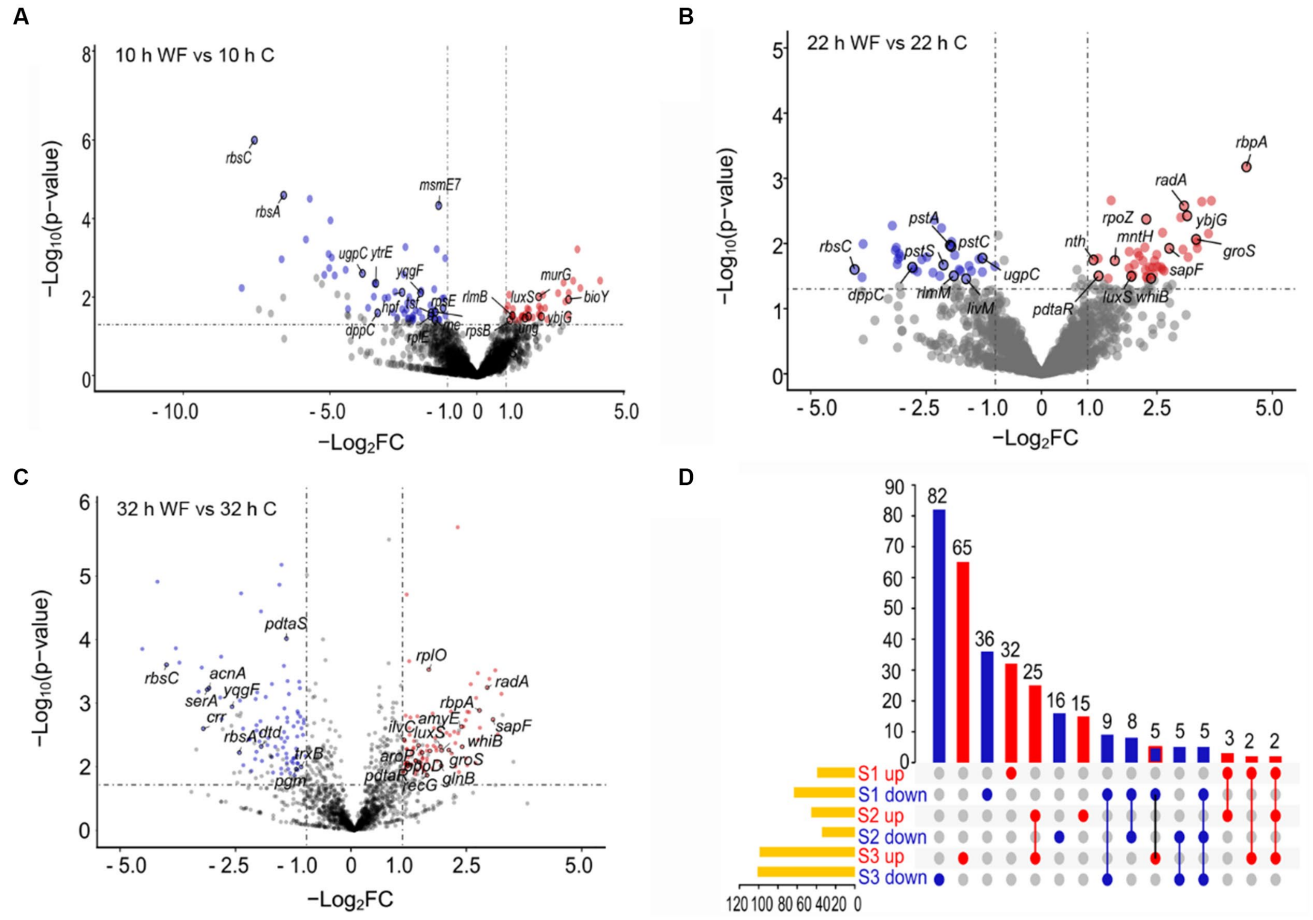
Transcripts profiling changes of *B. pseudocatenulatum* biofilm on wheat fibers groups (WF) and control groups **(C)** at three different stages including S1 (10 h), S2 (22 h), and S3 (32 h). **(A)** The volcano plot of S1 showing the DEGs between 10 h WF and 10 h C. Red points represent upregulated DEGs and blue points represent downregulated DEGs. WF: the biofilm on wheat fibers groups. C: the control group without wheat fibers. **(B)** The volcano plot of S2 showing the DEGs between 22 h WF and 22 h C. **(C)** The volcano plot of S3 showing the DEGs between 32 h WF and 32 h C. -Log_2_FC: -Log_2_(fold change). **(D)** The UpSet plot representation of the number of non-redundant DEGs at the three stages. The numbers upon red columns indicated the number of upregulated non-redundant DEGs. The numbers upon blue columns indicated the number of downregulated non-redundant DEGs.

To describe main gene changes during biofilm formation, we divided the *B. pseudocatenulatum* biofilm formation process into three stages and performed transcriptome analysis on specified pairwise transcriptome comparisons. The early stage of biofilm formation named S1 (10 h WF vs. 10 h C), maturation stage named S2 (22 h WF vs. 22 h C), and biofilm dispersion named S3 (32 h WF vs. 32 h C). This transcriptome analysis produced 381 DEGs. 102 DEGs of them were belonged to S1 (39 upregulated, 63 downregulated) (Figure 2A), 79 DEGs to S2 (45 upregulated, 34 downregulated) (Figure 2B), and 200 DEGs to S3 (99 upregulated, 101 downregulated) (Figure 2C). Total 310 non-redundant DEGs were shown on the UpSet plot (Figure 2D). There were 68 (32 upregulated, 36 downregulated), 31 (15 upregulated, 16 downregulated), and 147 (65 upregulated, 82 downregulated) unique DEGs in S1, S2, and S3, respectively. Notably, *luxS* (Bps41|peg.1262, involved in the synthesis of AI-2) was upregulated 3.38, 3.85, and 3.44-fold in S1, S2, and S3, respectively.

**Figure 3:**
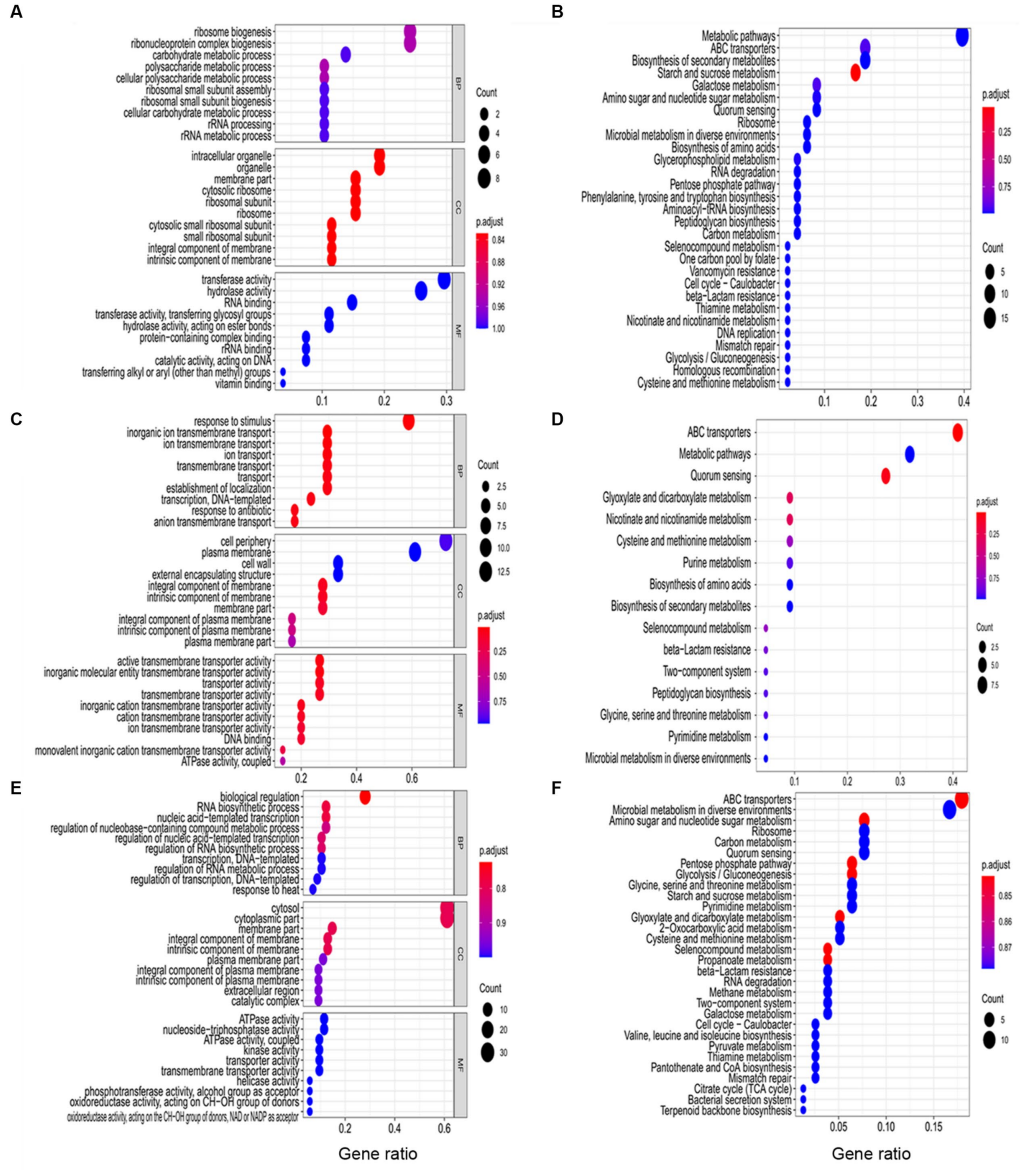
Functional analysis of DEGs at three different stages including S1 (10 h), S2 (22 h), and S3 (32 h). **(A)** The GO terms analysis and **(B)** KEGG analysis of DEGs in S1. **(C)** The GO terms analysis and **(D)** KEGG analysis of DEGs in S2. **(E)** The GO terms analysis and **(F)** KEGG analysis of DEGs in S3. BP: biological process. CC: cellular component. MF: molecular function.

The function of DEGs during *B. pseudocatenulatum* biofilm formation were analyzed by using GO and KEGG terms (Figure 3). DEGs in S1 (Figure 3A) were mainly associated with “polysaccharide metabolic process” (*glgC*, *glgP*, and *gtfA*), “peptide biosynthetic process” (*hpf*, *pheS*, *rplE*, *rpsB*, *rpsE*, and *tsf*), “cellular amino acid biosynthetic process” (*luxS* and *trpD*), “response to stress” (*ung*). Figure 3B shows the KEGG pathway at S1, “starch and sucrose metabolism” (*ams*, *bglB*, *crr*, *glgC*, *glgP*, and *gtfA*), “ABC transporters” (*amyC5*, *amyD3*, *bioY*, *dppC*, *msmE7*, *ugpC*, and *ytrE*), “amino sugar and nucleotide sugar metabolism” (*abfA1*, *crr*, *glgC*, and *pgl*), “phenylalanine, tyrosine and tryptophan biosynthesis” (*trpD* and *tyrA*), “peptidoglycan biosynthesis” (*murG* and *ybjG*), “QS” (*dppC*, *luxS*, Bps41|peg.1543, and Bps41|peg.980). GO terms of DEGs in S2 (Figure 3C) were including “response to stimulus” (*groS*, *mntH*, *nth*, *pdtaR*, *pstA*, *pstC*, *radA*, *rbpA*, *whiB*, and *ybjG*), “inorganic ion transmembrane transport” (*ctpE*, *mntH*, *pstA*, *pstC*, and *sstT*), “cell communication” (*luxS* and *pdtaR*), “vitamin metabolic process” (*pncA*), “positive regulation of biological process” (*rbpA*), “negative regulation of gene expression” (*whiB*). Meanwhile, Figure 3D shows that KEGG pathway of DEGs, including “ABC transporters” (*amyD3*, *dppC*, *livM*, *pstA*, *pstC*, *pstS*, *sapF*, *ugpC*, and Bps41|peg.247), “QS” (*dppC*, *livM*, *luxS*, *sapF*, Bps41|peg.1270, and Bps41|peg.1271), “Glyoxylate and dicarboxylate metabolism” (*pgp* and *glxK*), “nicotinate and nicotinamide metabolism” (*iunH2* and *pncA*), “two-component system” (*pstS*), “peptidoglycan biosynthesis” (*ybjG*), “glycine, serine and threonine metabolism” (*glxK*). GO terms of DEGs in S3 (Figure 3C) were including “response to stimulus” (*acnA*, *aroP*, *dtd*, *greA*, *groL*, *groS*, *grpE*, *pdtaR*, *pdtaS*, *radA*, *rbpA*, *recG*, *trxB*, and *whiB*), “oxidation–reduction process” (*acnA*, *fucO*, *ilvC*, *pgm*, *serA*, *trxB*, and *whiB*), “peptide biosynthetic process” (*rplI*, *rplO*, *rpmA*, and *rpsT*). The KEGG pathway (Figure 3F) included “ABC transporters” (*amyC5*, *amyD3*, *amyE*, *amyE*, *ecfA*, *glnQ*, *msmE*, *msmE7*, *pstS*, *sapF*, Bps41|peg.1139, Bps41|peg.1621, Bps41|peg.1741, and Bps41|peg.1861), “QS” (*luxS*, *oppD*, *sapF*, *secG*, Bps41|peg.1139, and Bps41|peg.1271), “glyoxylate and dicarboxylate metabolism” (*acnA*, *fucO*, *glxK*, and *pgp*), “two-component system” (*glnB*, *pstS*, and Bps41|peg.1679), “cysteine and methionine metabolism” (*ldh*, *luxS*, and *metB*), “valine, leucine and isoleucine biosynthesis” (*ilvC* and *ilvN*).

A PPI network between DEGs had 55 nodes with 140 links, and 4 clusters (Supplementary Figure S1). There were 10 DEGs in cluster 1 and mainly related to translation and peptide biosynthetic processes. Cluster 2 was associated with polysaccharide metabolic process. Cluster 3 was about QS, *rbpA* upregulated 21.69-fold in S2 and *secG* was upregulated 2.54-fold in S3, *pstA, pstC* and *phoU* in cluster 4 (involved in phosphate import) were downregulated during biofilm formation. In addition, *radA* (plays a role in repairing DNA breaks) was upregulated 2.02, 8.51, and 6.73-fold, respectively. *lexA* involved in the response to DNA damage (SOS response), was downregulated 2.85-fold in S3. The result indicated that the DNA damage of biofilm cells was reduced in this stage. The interaction of DEGs evidenced that peptide biosynthetic and polysaccharide metabolic process were important during biofilm formation. There were only 55 genes of redundant DEGs involved in PPI network, meaning that the most interactions between DEGs related to biofilm formation were still unclear.

### 3.3. Thirteen modules identified and two of them positively correlated to biofilm formation rate

**Figure 4:**
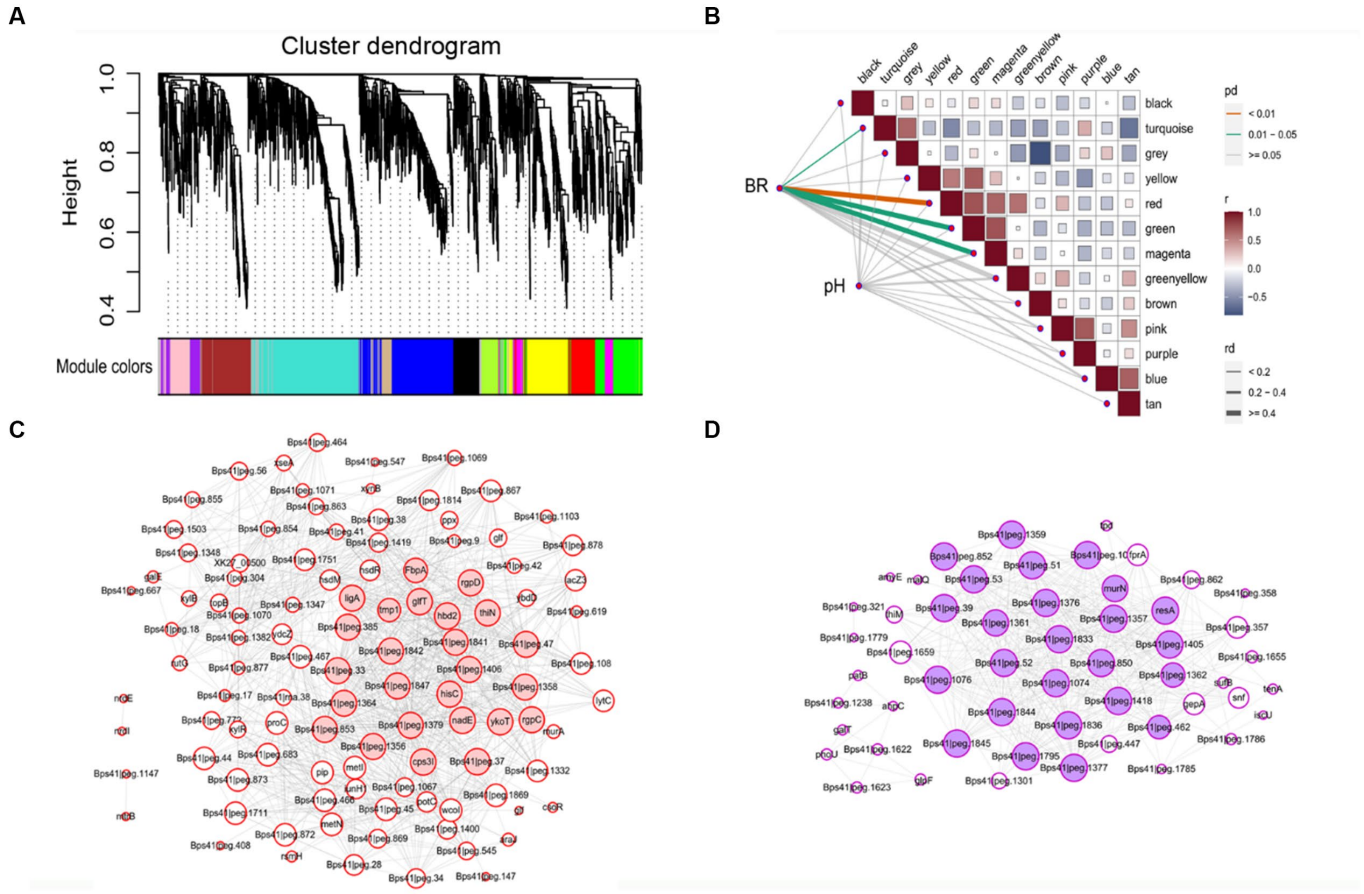
The WGCNA of expressed genes during *B. pseudocatenulatum* biofilm formation. **(A)** Hierarchical cluster tree of co-expressed genes. Each of the 1933 genes is represented by a leaf in the tree. The lower panel shows modules in designated colors, such as “Blue”, “Pink”, “Turquoise,” and others. Note that module “Gray” is for unassigned genes. **(B)** Module-pH/BR correlations and corresponding *p*-values, BR: biofilm formation rate. **(C)** Co-expression network analysis of red module. **(D)** Co-expression network analysis of magenta module. The size of the node circle is positively correlated to the number of interacting gene partners. The genes filled in red present the hub genes of red module, and genes filled in magenta present the hub genes of magenta module.

A scale-free gene co-expression network was performed with 1933 transcript genes to identify the genes related to biofilm formation. These genes were classified to 13 WGCNA modules (Figure 4A). Analysis of the module-trait relationships (Figure 4B) revealed that BR was positively correlated to 114 genes in the red module (*r* = 0.70, *p* < 0.01) and 69 genes in the magenta module (*r* = 0.60, *p* < 0.01). *glfT* in the red module was related to cellular polysaccharide metabolic process. *rplO* in the magenta module was related to peptide biosynthetic process. *csoR*, *ligA*, and *rutG* in the red module and *dtd* in magenta module were associated with response to stimulus. *galE*, *glf*, *glf*, *murA*, and *xynB* in the red module and *galT* in magenta module were related to amino sugar and nucleotide sugar metabolism. *metI*, *metN*, *potC*, and *rgpD* in the red module and *amyE* in magenta module were related to ABC transporters. *mtrB* in the red module was related to a two-component system. *ydcZ* in the red module was related to QS.

Some hub genes were identified in the red module, including *rgpD* (ABC transporter, ATP-binding protein), *glfT* (glycosyltransferase like family 2), *ykoT* (glycosyl transferase family 2), *rgpC* (transport permease protein), and Bps41|peg.1842 (glycosyltransferase like family 2) (Figure 4C). The key hub genes were *resA* (Type III restriction enzyme, res subunit), *murN* (Psort location Cytoplasmic), Bps41|peg.1844 (GDSL-like Lipase/Acylhydrolase family), Bps41|peg.1076 (Helicase conserved C-terminal domain), and Bps41|peg.1362 (psort location CytoplasmicMembrane) in the magenta module (Figure 4D). It can be seen that co-expressed gene modules have different functions in the process of biofilm formation.

### 3.4. Peptide biosynthetic process and EPS production involved in biofilm formation

**Figure 5:**
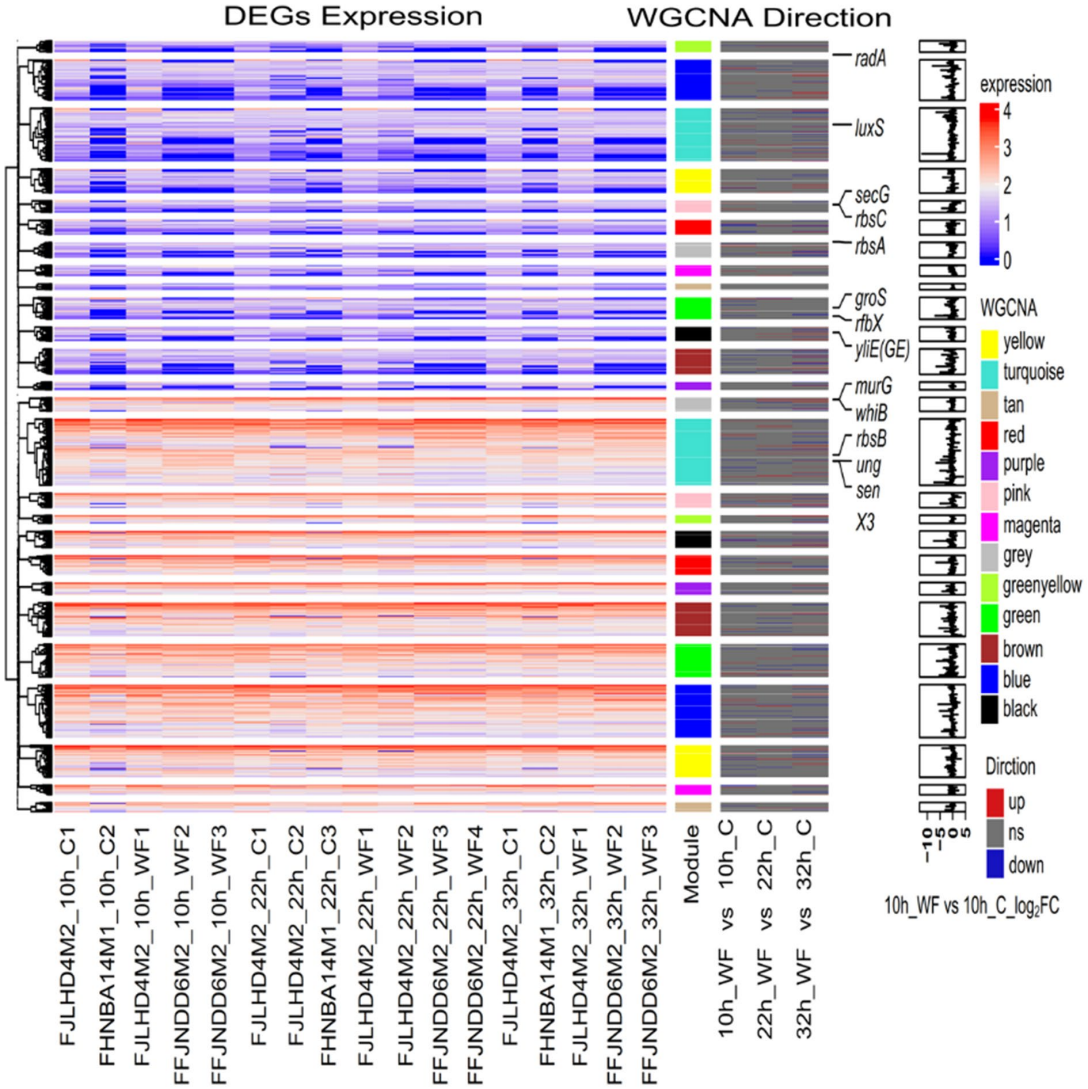
Heatmap and hierarchical clustering of genes expression during biofilm formation. The left heat map shows the genes expression in different samples. Red indicates a higher gene expression level, and blue indicates a lower gene expression level. The color from red to blue indicates log10 (FPKM+1) from high to low. FPKM: fragments per kilobase of exon per million mapped fragments. The middle heat map shows the three directions of genes, upregulated, downregulated or ns (no significance). The right bar graph is the differential expression log_2_ (fold change) of genes in S1 stage (10 h WF vs. 10 h C).

Using known genes involved in biofilm formation might predict whether new genes are also involved in biofilm processes, as these genes with similar expression patterns may have similar functions. Based on the WGCNA modules, 1933 genes were classified into 13 clusters (Figure 5). The Turquoise module contained 104 DEGs. There were 17 genes (including *aroG*, *fadD*, *glxR*, *luxS*, *oppD*, *sapF*, *secG*, and *tsaD*) in turquoise module related to QS; 14 genes (*alaA*, *arc*, *dnaK*, *frc*, *groS*, *grpE*, *mntH*, *nudC*, *pdtaR*, *radA*, *recF*, *ruvC*, *ung*, and *uvrA*) associated with cellular response to stimulus; 5 genes (*dnaA*, *glnB*, *glxR*, Bps41|peg.129, and Bps41|peg.1679) associated with two-component system; *alaA*, *ilvC*, and *ilvN* associated with valine, leucine and isoleucine biosynthesis. There were 30 genes in the yellow module associated with peptide biosynthetic process. *aroG*, *lepB*, *secE*, *ydcZ*, and *yidC* associated with QS. *rfbX* (Bps41|peg.1370, polysaccharide biosynthetic process) and Bps41|peg.1372 (acyltransferase family) in yellow module associated with EPS production. *galE* (belongs to the NAD(P)-dependent epimerase dehydratase family) in the red module was downregulated 1.16-fold in S1, and then upregulated 1.11-fold in S2. However, the expression of *galE* was high during biofilm formation (over 1,000 in S1). Interestingly, *zwf* (encoding glucose-6-phosphate dehydrogenase) in blue module and *opcA* (glucose-6-phosphate dehydrogenase subunit) in greenyellow module were upregulated 2.34 and 2.30-fold in S1, respectively.

### 3.5. Metabolites profiling during biofilm formation

**Figure 6:**
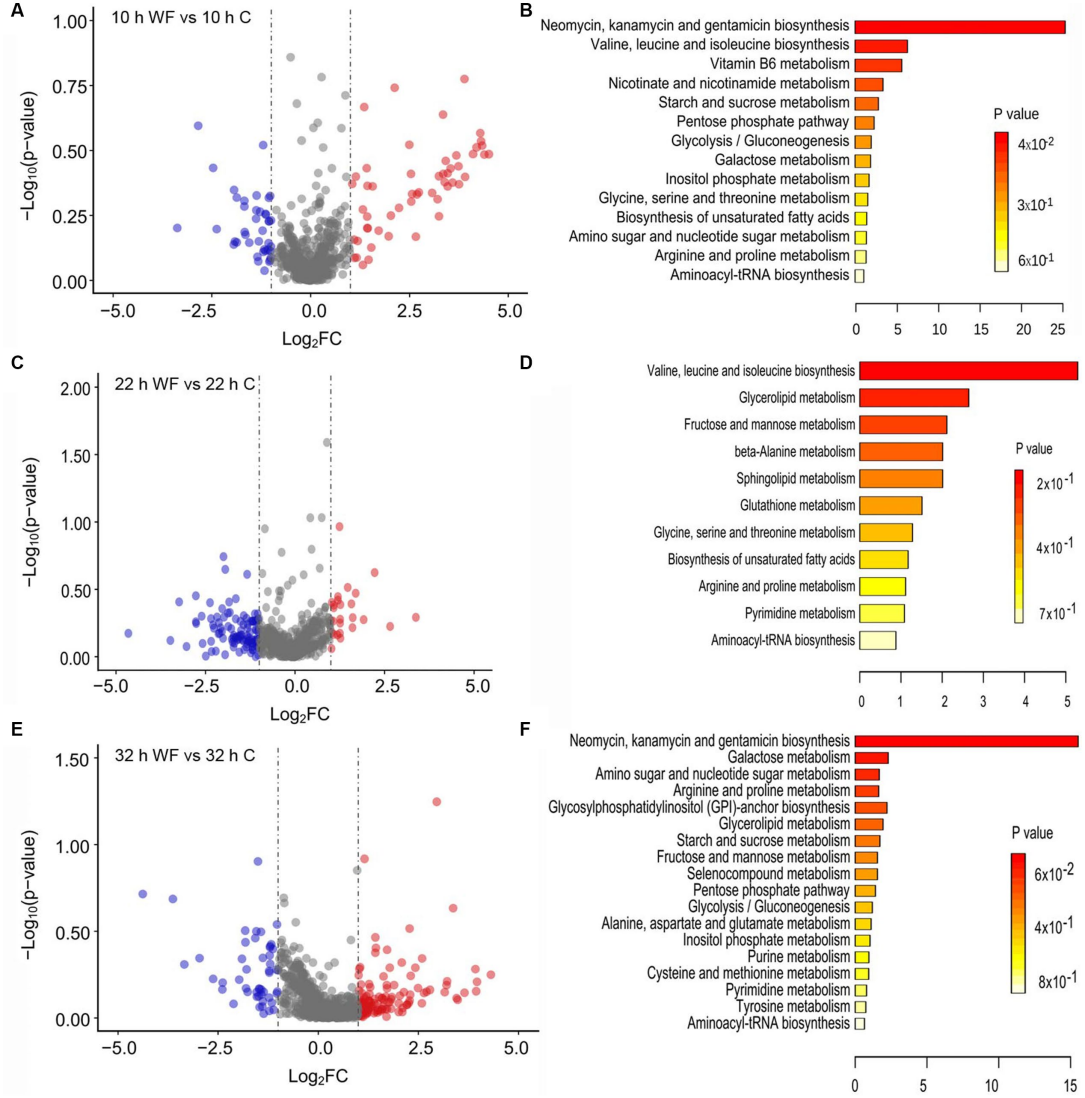
Metabolites profiling changes and enrichment pathway analysis during biofilm formation on wheat fibers (WF) and control (C) groups. **(A,B)** The volcano plot showing the upregulated (red points) and downregulated (blue points) metabolites and enrichment pathway analysis of extracellular metabolites in S1 (10 h WF vs. 10 h C). **(C,D)** The volcano plot showing the upregulated and downregulated metabolites and enrichment pathway analysis of extracellular metabolites in S2 (22h WF vs. 22 h C). **(E,F)** The volcano plot showing the upregulated and downregulated metabolites and enrichment pathway analysis of intracellular metabolites in S3 (32 h WF vs. 32 h C). -Log_2_FC: -Log_2_(fold change).

To identify the key metabolites involved in biofilm, LC–MS data analysis of biofilm and planktonic metabolites at different biofilm formation stage was conducted. There were 94 differentially expressed extracellular metabolites (53 upregulated, 41 downregulated) in S1 (Figure 6A). Figure 6B shows that the enrichment pathway of these extracellular metabolites in S1. L-threonine was upregulated 2.96-fold (glycine, serine and threonine metabolism; valine, leucine and isoleucine biosynthesis; aminoacyl-tRNA biosynthesis), 4-acetamidobutanoic acid (arginine and proline metabolism) was upregulated 2.07-fold; glucose 6-phosphate was downregulated 2.30-fold (starch and sucrose metabolism; glycolysis/gluconeogenesis; pentose phosphate pathway; galactose metabolism; inositol phosphate metabolism; amino sugar and nucleotide sugar metabolism), and pyridoxal (vitamin B6 metabolism) was downregulated 2.58-fold, niacinamide (nicotinate and nicotinamide metabolism) was downregulated 2.03-fold. In S2, there was 26 upregulated and 108 downregulated extracellular metabolites (Figure 6C). Figure 6D shows that the enrich pathway of these extracellular metabolites in S2: L-threonine was upregulated 2.41-fold, glyceraldehyde was downregulated 2.10-fold (glycerolipid metabolism; fructose and mannose metabolism), and spermine was downregulated 3.58-fold (beta-alanine metabolism; glutathione metabolism; arginine and proline metabolism).

There were 130 upregulated and 50 downregulated intracellular metabolites in S3 (Figure 6E). The enriched pathways of intracellular metabolites (Figure 6F) were including: amino sugar and nucleotide sugar metabolism (glucose-6-phosphate; uridine diphosphategalactose); arginine and proline metabolism (4-hydroxyproline, 4-acetamidobutanoic acid); cysteine and methionine metabolism (L-cystine); and aminoacyl-tRNA biosynthesis (L-alanine).

### 3.6. Amino acids, organic acids, and sugar were main metabolites during biofilm formation

**Figure 7:**
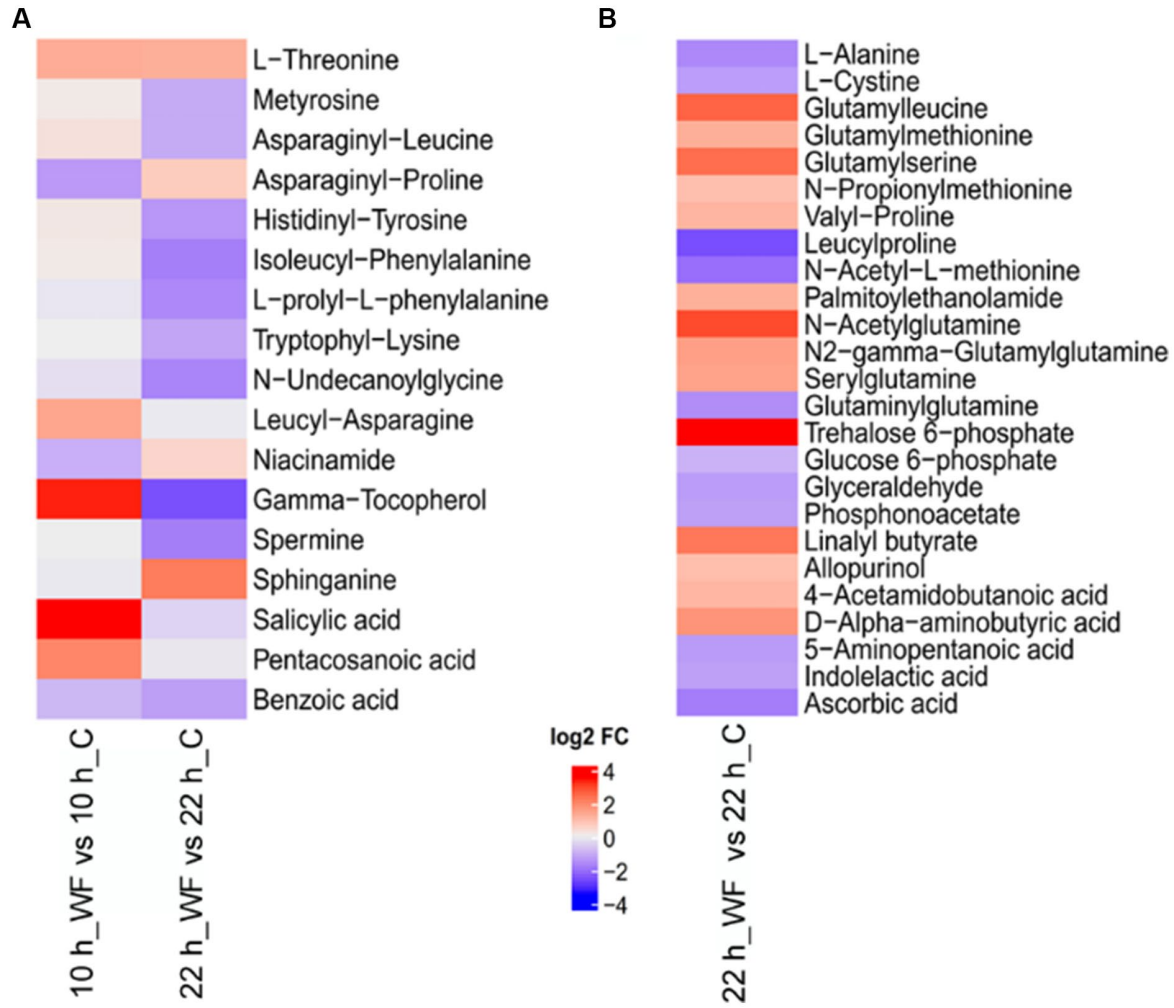
Heatmap of key metabolites during biofilm formation on wheat fibers (WF) and control (C) groups. **(A)** Extracellular metabolites and **(B)** intracellular metabolites. Red represents upregulated and blue represents downregulated.

MetaboAnalyst5.0 was used to search the function of differentially expressed metabolites. Extracellular metabolites mainly included amino acids and organic acids (Figure 7A). L-threonine, leucyl-asparagine, gamma-tocopherol, salicylic acid, pentacosanoic acid were upregulated 2.96, 2.69, 10.18, 11.05, and 4.08-fold in S1, respectively. Sphinganine was upregulated 4.67-fold in S2. Figure 7B shows the main differentially expressed intracellular metabolites (14 upregulated, 11 downregulated) in S2. The top ten upregulated metabolites were: trehalose-6-phosphate, N-acetylglutamine, glutamylleucine, glutamylserine, linalyl butyrate, D-alpha-aminobutyric acid, N2-gamma-glutamylglutamine, serylglutamine, glutamylmethionine, and valyl-proline.

## 4. Discussion

In this study, we collected samples from three *B. pseudocatenulatum* strains (FJLHD4M2, FFJNDD6M2, FHNBA14M1) under biofilm and planktonic conditions during fermentation and analyzed the main DEGs at each stage that included: S1 (10 h WF vs. 10 h C), the early stage of biofilm formation; S2 (22 h WF vs. 22 h C), the stage of biofilm maturation; and S3 (32 h WF vs. 32 h C), the stage of biofilm dispersion. Transcriptomic analysis indicated that genes associated with stress response, QS, polysaccharide metabolic process may be involved in biofilm formation. Metabonomic analysis indicated that amino acids and amides (L-threonine, leucyl-asparagine, N-acetylglutamine, glutamylleucine, glutamylserine, D-alpha-aminobutyric acidvalyl-proline, N2-gamma-glutamylglutamine, serylglutamine, and glutamylmethionine); organic acids, alcohols and esters (salicylic acid, pentacosanoic acid, linalyl butyrate, and sphinganine); and sugar (trehalose-6-phosphate, uridine diphosphategalactose, and uridine diphosphate-N-acetylglucosamine) were main metabolites during biofilm formation. Uridine diphosphate galactose (UDP-galactose) is an intermediate in the production of polysaccharides, which is important in nucleotide sugars metabolism (Vert et al., 2012).

EPS is crucial in the process of biofilm formation. Usually, strengthening the synthesis of basic sugar units can increase the yield of EPS. The *glgC* is a gene encoding glucose-1-phosphate adenylate transferase, which can redirect carbon sources into the EPS synthesis pathway. In addition, overexpression of *gpi* encoding glucose-6-phosphate isomerase can also enhance glucose metabolism and accumulate sugar monomers. *Synechocystis* sp. PCC 6803, which was knocked out of *glgC* and overexpressed with *gpi* (▲*gpi*-∆*glgC*), produced the highest content of EPS (Velmurugan and Incharoensakdi, 2021). *Zwf*, a gene encoding glucose-6-phosphate dehydrogenase (G6PD), increases EPS production by catalyzing conversion of glucose 6-phosphate into gluconate 6-phosphate (Guo et al., 2015). The deletion mutation in *zwf* reduced the motility of bacteria and EPS production. The deletion mutagenesis in *zwf* also altered the transcript level of key genes in diffusible signal factor signaling network. Interestingly, in our study, the biofilm rate was exceeded 35% in S1 (Figure 1). *glgC* was downregulated 2.73-fold in S1. *zwf* and *opcA* were upregulated 2.34 and 2.30-fold in S1 (Figure 2). However, glucose 6-phosphate was downregulated 2.30-fold in S1 (Figure 6). The *galE* in the red module was downregulated 1.16-fold in S1, and then upregulated 1.11-fold in S2. Uridine diphosphategalactose, and uridine diphosphate-N-acetylglucosamine were upregulated 4.03, 2.38-fold in S2. The enzyme, *galE*, overexpression led to an increased capacity of *Thermus thermophilus* HB27 biofilm formation, suggesting that the *galE* gene is important for biofilm formation as it participates in epimerization of uridine diphosphate galactose and uridine diphosphate N-acetylgalactosamine for extracellular polysaccharide biosynthesis (Niou et al., 2009). These results indicated that EPS was produced to promote the biofilm formation in S1. In mixed-species biofilm, the *galE* gene of *B. bifidum* also showed a significant increase in expression, which led the biofilm mass of mixed-species increased (Sadiq et al., 2021). Hence one can see that *galE* is an important gene for improving biofilm formation, not only for mono-species biofilm but also for mixed-species biofilm. And this positive relation is common.

Metabonomic analysis indicated that dipeptides and organic acids were main metabolites for *B. pseudocatenulatum* biofilm formation. In bacterial biofilms, organic acids are mainly produced by the fermentation of sugars and can reduce the pH values (Suzuki et al., 2020). Amino acids generate a large amount of energy during the gluconeogenesis process. The formation of biofilms requires cell adhesion, surface regulation, and EPS production, all of which are energy intensive processes. As the biofilm of *Bifidobacterium* maturing, the abundance of amino acids and energy production decrease (Liu et al., 2021b). Compared with oligotrophic conditions, bacteria formed biofilms in eutrophic systems with a shorter lag period and increased growth rates (Saville et al., 2011; Flemming et al., 2016; Niederdorfer et al., 2017). Therefore, the accumulation of amino acids may attribute to the production of EPS. Increased EPS production also reflects a highly adaptive response of biofilms to environmental stress factors (Liu et al., 2021a).

In short, *B. pseudocatenulatum* can form biofilms on wheat fibers. Transcriptomic analysis exhibited that genes related to stress response and polysaccharide metabolic process may be included in biofilm formation. In addition, 17 WGCNA modules were identified and two of them positively correlated to BR. Metabonomic analysis indicated that amino acids, amides, organic acids, alcohols, esters, and sugar (trehalose-6-phosphate, uridine diphosphategalactose, uridine diphosphate-N-acetylglucosamine) were main metabolites during biofilm formation. These results showed that stress response, QS, and EPS production were essential during *B. pseudocatenulatum* biofilm formation. Our study offers valuable insights into the molecular mechanisms underpinning biofilm formation in *B. pseudocatenulatum*. The fundamental significance lies in enhancing our understanding of bacterial biofilms, particularly in the context of *Bifidobacterium*, an area that has been relatively under-explored. By identifying genes and metabolites crucial for biofilm formation, we pave the way for targeted interventions that can modulate these processes. Practically, these findings can influence the development of probiotic formulations, where biofilm formation can be a desirable trait for enhanced gut colonization and resilience. Moreover, understanding the mechanisms of biofilm formation can also assist in addressing challenges associated with biofilm-related infections or industrial processes where biofilms play a role.

## 5. Conclusion

A multi-omics analysis using transcriptomics and metabolomics was conducted on the biofilm formation process of *B. pseudocatenulatum* found that the formation of biofilm was related to stress response, QS, and EPS production. However, further exploration is needed on the key substances, key genes, and key mechanisms involved in the formation of *B. pseudocatenulatum* biofilm.

## Data availability statement

The datasets presented in this study can be found in online repositories. The names of the repository/repositories and accession number(s) can be found at: https://www.ncbi.nlm.nih.gov/, PRJNA738670.

## Author contributions

TZ, ZL, HW, HZ, HL, and WL contributed to conceptualize and provided methodology to the study. TZ and ZL wrote the original draft. TZ, ZL, HW, HZ, HL, WL, and JZ reviewed and edited the manuscript. WL and JZ provided funding and resources. All authors contributed to the article and approved the submitted version.

## Footnotes

^1^ https://www.ncbi.nlm.nih.gov/bioproject/PRJNA738670/

^2^ https://www.metaboanalyst.ca
