# Supplementary material for: Multi-omics analysis reveals genes and metabolites involved in Bifidobacterium pseudocatenulatum biofilm formation
Source: Front Microbiol. 2023 Nov 9;14:1287680. doi: 10.3389/fmicb.2023.1287680 (PMC10666050; doi:10.3389/fmicb.2023.1287680)
Supplement: Supplementary file 1 [file Data_Sheet_1.PDF]

Multi-omics analysis reveals genes and metabolites involved in *Bifidobacterium pseudocatenulatum* biofilm formation

Ting Zhang[1,2], Zongmin Liu[1,2], Hongchao Wang[1,2], Hao Zhang[1,2,3], Haitao Li[1,2], Wenwei Lu[1,2,4] and Jinlin Zhu[1,2*]

[1]State Key Laboratory of Food Science and Resources, Jiangnan University, Wuxi 214122, China, [2]School of Food Science and Technology, Jiangnan University, Wuxi 214122, China, [3](Yangzhou) Institute of Food Biotechnology, Jiangnan University, Yangzhou 225004, China, [4]National Engineering Research Center for Functional Food, Jiangnan University, Wuxi 214122, China.

*** Correspondence:**
Jinlin Zhu
wx_zjl@jiangnan.edu.cn

Supplementary Figures

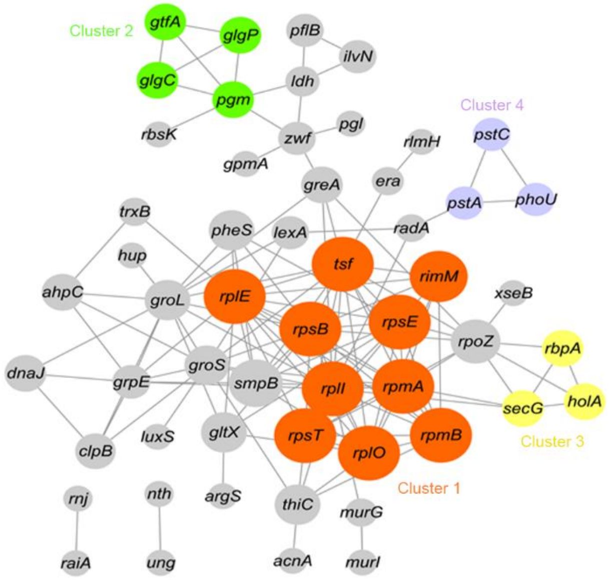

FIGURE S1. *B. pseudocatenulatum* biofilm formation protein–protein interaction (PPI) network. Each cluster is a set of highly-connected nodes and the size of the node was determined by MCODE degree.

Supplementary Tables

TABLE S1 The biofilm formation rate of *B. pseudocatenulatum* at different formation times.

Strains	Time (h)	BR (%)
FJHD4M2	10	35.18
	22	46.56
	32	61.03
FFJNDD6M2	10	58.90
	22	78.96
	32	49.38
FHNBA14M1	10	2.17
	22	4.76
	32	4.31
